# Cardiovascular toxicity in testicular germ cell tumor survivors

**DOI:** 10.3389/fonc.2025.1654063

**Published:** 2025-08-13

**Authors:** Zuzana Orszaghova, Beata Mladosievicova, Michal Mego, Michal Chovanec

**Affiliations:** ^1^ 2^nd^Department of Oncology, Faculty of Medicine, Comenius University and National Cancer Institute, Bratislava, Slovakia; ^2^ Department of Clinical Pathophysiology, Faculty of Medicine, Comenius University, Bratislava, Slovakia

**Keywords:** cardiotoxicity, cancer treatment, late toxicity, testicular cancer, germ cell tumor, survivorship

## Abstract

**Introduction:**

Testicular germ cell tumors (TGCT) are highly curable malignancies, with excellent survival rates largely attributable to advances in cancer treatment. Consequently, there is a growing population of long-term TGCT survivors whose life expectancy approaches that of the general population. However, these survivors may experience acute and late adverse effects of cancer treatment, with cardiovascular toxicity being among the most serious and potentially life-threatening.

**Methods:**

This narrative review synthesizes current evidence on cardiovascular toxicity in testicular cancer survivors, including clinical manifestations, pathophysiology of cisplatin-induced cardiovascular damage, additional adverse effects of radiotherapy, and prevalence of traditional cardiovascular risk factors. Key clinical guidelines, observational studies, and experimental findings were analyzed to identify trends, knowledge gaps, and opportunities for improving survivorship care.

**Results:**

Multiple studies consistently demonstrate an increased risk of cardiovascular disease (CVD) among TGCT survivors, particularly following cisplatin-based chemotherapy. Common clinical manifestations include myocardial infarction, angina pectoris, cerebrovascular events, thromboembolism, and heart failure. The highest risk occurs within the first year post-treatment but may persist or recur even after a decade. Cisplatin-induced cardiovascular toxicity involves vascular injury - characterized by endothelial dysfunction, oxidative stress, and prothrombotic state - and myocardial damage driven by oxidative stress, inflammation, and apoptosis. Furthermore, TGCT survivors exhibit a higher prevalence of traditional cardiovascular risk factors, such as smoking, hypertension, dyslipidemia, diabetes, and obesity, contributing to the overall elevated CVD risk.

**Discussion:**

There is an urgent need for a structured, long-term survivorship care model for TGCT survivors. Cardiovascular risk assessment and prevention should be central components, especially in survivors treated with cisplatin-based chemotherapy. Early detection of treatment-related toxicities, combined with lifestyle interventions and regular monitoring, is essential. Future research should focus on elucidating molecular mechanisms of cardiovascular toxicity, validating TGCT survivor-specific screening tools, identifying early biomarkers of cardiac injury, and exploring pharmacologic and behavioral interventions.

**Conclusion:**

Protecting cardiovascular health in TGCT survivors requires a proactive, personalized, and multidisciplinary approach. Integrating cardiometabolic monitoring, risk factor modification, and tailored follow-up strategies into survivorship care is vital. Focused research and clinical attention are needed to ensure that the long-term success of cancer treatment is not compromised by preventable cardiovascular disease.

## 1 Introduction

In both Europe and United States, cardiovascular disease (CVD) and cancer are among the leading causes of morbidity and mortality (1, 2). Current evidence indicates a bidirectional relationship between these conditions, driven by shared risk factors and overlapping pathophysiological mechanisms (3).

Testicular germ cell tumors (TGCT) are the most common solid malignancies in men aged 20 to 40 years, with a globally rising incidence in recent decades (4, 5). TGCTs are highly curable, with an outstanding > 95% 5-year survival rate, largely attributable to advances in cancer treatment, especially surgery and cisplatin-based chemotherapy (6–8).

Successful cancer treatment has led to a growing number of long-term TGCT survivors whose life expectancy is comparable to those of the healthy population (9). However, these survivors may experience a range of acute and late adverse effects from cancer treatment, including secondary malignancies, an increased risk of cardiovascular disease, pulmonary toxicity, nephrotoxicity, ototoxicity and neurotoxicity, hypogonadism, infertility, and sexual dysfunction. In addition, TGCT survivors may experience psychosocial problems such as depression, anxiety, sleeping disturbances, post-traumatic stress disorder (PTSD), and cognitive dysfunction. All of these adverse effects can contribute to a reduced quality of life. Secondary malignancies and cardiovascular toxicity are the most serious, and potentially life-threatening consequences, as well as the most common causes of mortality in testicular cancer survivors (10).

Cardiovascular toxicity may occur at any time, from days after the first dose of cisplatin to several years following the completion of treatment. Acute cardiovascular events (such as acute vasospasm, thromboembolism, arrhythmias) typically manifest within the first year after exposure to chemotherapy. Late cisplatin-related cardiovascular toxicity is diagnosed beyond 12 months after completing the cardiotoxic treatment, and includes mainly ischemia, myocardial infarction, cardiac dysfunction/heart failure, hypertension, and hyperlipidemia.

The increased risk of CVD in this population may result not only from the adverse cardiovascular effects of anticancer therapies, but also from preexisting cardiovascular risk factors and to advanced cancer itself (11–14).

In this review, we summarize current evidence on cardiovascular toxicity in testicular cancer survivors, including its clinical manifestations, the pathophysiology of cisplatin-related cardiovascular toxicity, and the prevalence of cardiovascular risk factors. We conducted a narrative review of the literature, focusing on clinical and epidemiological studies published in peer-reviewed journals. Our aim is to highlight key findings and emphasize the critical need for increased awareness and integration of cardiovascular health into long-term survivorship care. A comprehensive literature search was conducted using PubMed and related databases up to June 2025. Keywords included “testicular cancer,” “germ cell tumor,” “cancer treatment,” “cisplatin,” “cardiovascular toxicity,” and “cardiovascular disease.” Studies were selected based on their relevance to cardiovascular outcomes in testicular cancer survivors, focusing on clinical manifestations, risk factors, and population-based data. Both original research articles and relevant reviews published in English were included. Articles not available in English or lacking sufficient clinical data were excluded.

## 2 Cardiovascular disease in testicular cancer survivors

### 2.1 Clinical manifestations of cardiovascular toxicity

Cardiovascular toxicity in TGCT survivors encompasses a spectrum of clinical manifestations of cardiac and vascular damage, ranging from asymptomatic ECG changes, cardiac conduction system abnormalities, and alterations in cardiac function or structure, to life-threatening cardiovascular events (CVE) such as myocardial infarction, heart failure, thromboembolism, and cerebrovascular accidents. However, subclinical electrophysiological, structural, and functional cardiovascular abnormalities may not necessarily progress to clinical CVE (15, 16).

Although cardiac toxicity associated with cisplatin is relatively rare, some reports have described cardiac events suggestive of vasospasm, ischemia, hypertension, decreased diastolic and/or systolic function, myocarditis, pericarditis, myocardial infarction, stroke, and heart failure (15, 17, 26, 27). In addition, cisplatin has occasionally been reported to cause cardiac arrhythmias, including supraventricular tachycardia, atrial fibrillation, ventricular arrhythmias, sinus bradycardia, left bundle branch block, atrioventricular block, and, infrequently, complete atrioventricular block (28). Cisplatin is also associated with an increased risk of thromboembolic events, such as arterial thrombosis, deep venous thrombosis, and pulmonary embolism (16, 29).

### 2.2 Risk of cardiovascular disease

Over the years, multiple studies have demonstrated increased risk of CVD among survivors treated for testicular cancer, particularly those who received cisplatin-based chemotherapy (16–24). In contrast, the risk of CVD and CVD-related mortality in patients with clinical stage I disease treated with orchiectomy alone appears to be comparable to that of the general population (25). Studies reporting the risk of CVD in TGCT survivors are summarized in **Table 1**.

**Table 1:** Summary of studies reporting cardiovascular disease risk in testicular germ cell tumor survivors.

Author (Year)	N	Treatment years	Median follow-up, years (range)	Median age at follow-up, years (range)	Cardiovascular toxicity	Incidence of CVD, N (%)	Risk of CVD
Meinardi et al. (2000) (17)	87	Before 1987	14 y (10-20)	41 y (30-50)	MI and AP with confirmed myocardial ischemia	5 (6%)	7.1 (95% CI, 1.9–18.3) ^a^
Huddart et al. (2003) (18)	992	1982 - 1992	10.2 y (0-20.3)	44 y (23-78)	Any CVE	68 (6.8%)	CT: RR 2.59 (95% CI, 1.15–5.84) RT: RR 2.40 (95% CI, 1.04–5.45) CTRT: RR 2.78 (95% CI, 1.09–7.07) ^b^
Van den Belt-Dusebout et al. (2006) (19)	2,512	1965 - 1995	18.4 y (5-38.4)	38.3 y (seminoma); 28.1 y (nonseminoma) ^c^	MI, AP, and HF	357 (14.2%)	PVB: HR 1.9 (95% CI, 1.2-2.9) BEP: HR 1.5 (95% CI, 1.0-2.2) ^b^
Van den Belt-Dusebout et al. (2007) (20)	2,707	1965 - 1995	17.6 y (NR)	38.3 y (seminoma); 28.2 y (nonseminoma) ^c^	MI, AP, and HF	357 (13.2%)	CT: HR 1.7 (95% CI, 1.1-2.5) RT: HR 1.2 (95% CI, 0.9-1.7) ^b^
Haugnes et al. (2010) (21)	784	1980 - 1994	19 y (13-28)	51 y (31-69)	MI and AP	52 (5.6%)	BEP: HR 5.7 (95% CI, 1.9-17.1) RT: HR 2.1 (95% CI, 0.78-5.4) CTRT: HR 5.3 (95% CI, 1.5-18.3)
Fung et al. (2015) (22)	15,006	1980 - 2010	7.9 y (surgery only); 6.5 y (CT)	NR	CVD mortality	NA	CT: SMR 1.36 (95% CI, 1.03-1.78) ^d^
Lauritsen et al. (2020) (16)	5,185	1984 - 2007	15.8 y (9.8-22.0)	NR	MI	NA	BEP (1^st^ year): HR 6.3 (95% CI, 2.9-13.9) ^d^
Lubberts et al. (2023) (24)	4,748	1976 - 2007	16.1 y (0-38.5)	NR	MI or CAD without infarction	272 (5.7%)	CT: HR, 1.9 (95% CI, 1.1-3.1) CTRT: HR 2.5 (95% CI, 1.2-5.1) ^b^

AP, angina pectoris; BEP, bleomycin, etoposide, cisplatin; CAD, coronary artery disease; CT, chemotherapy; CTRT, chemotherapy and radiotherapy, CVD, cardiovascular disease; CVE, cardiovascular event; HF, heart failure; HR, hazard ratio; MI, myocardial infarction; NA, not applicable; NR, not reported; PVB, cisplatin, vinblastine, bleomycin; RR, relative risk; RT, radiotherapy; SMR, standard mortality ratio; y, years.

a Observed-to-expected ratio; compared with general male Dutch population.

b Compared with surgery/orchiectomy only group.

c Both are median ages at diagnosis; median ages at follow-up were not reported.

d Compared to general population.

The risk of cisplatin-induced CVD appears to be highest within the first year after treatment but may persist or increase again after several years, suggesting a biphasic pattern of cardiovascular risk (16, 19, 21). Late cisplatin-related cardiovascular toxicity can occur more than ten years post-treatment, leading to atherogenesis, thrombosis, and premature vascular aging (30–32).

It remains unclear whether cardiovascular events in TGCT survivors are solely attributable to cisplatin, other anticancer agents or the underlying malignancy itself (11). Current evidence suggests that testicular cancer, compared to other malignancies such as pancreatic, gastric, or lung cancer, carries a relatively low intrinsic risk of cancer-associated thrombosis. Rather, the increased cardiovascular risk appears to result from cancer treatments, treatment-induced metabolic changes, and both preexisting and therapy-related cardiovascular risk factors (13). Importantly, thrombotic risk is also influenced by disease stage and patients with advanced TGCT, where orchiectomy alone is insufficient, exhibit a higher incidence of thrombotic and cardiovascular events (12–14). This helps explain the apparent discrepancy: while orchiectomy in early-stage TGCT does not increase CVD risk, the need for systemic treatment in advanced-stage disease introduces additional cardiovascular burden.

### 2.3 Population-based studies

A Dutch study (17) reported major CVE in 5 (6%) of 87 TGCT survivors (aged 30–42 years; 9–16 years post-chemotherapy). Two experienced myocardial infarctions (one of which was fatal), and three developed angina pectoris with confirmed myocardial ischemia. Compared to the general Dutch male population, the observed-to-expected ratio for coronary artery disease was 7.1 (95% CI, 1.9–18.3). In addition, one patient aged 41 experienced a cerebrovascular accident 11 years after chemotherapy. Huddart et al. (18) observed 68 CVE (including 18 deaths) among 992 TGCT patients after a median follow-up of 10.2 years. Compared to the orchiectomy-only group, the risk of developing CVD was more than two-fold higher following chemotherapy (relative risk [RR] 2.59; 95% CI, 1.15–5.84; *p* = 0.022), radiotherapy (RR 2.40; 95% CI, 1.04–5.45; *p* = 0.036), and chemotherapy + radiotherapy (RR 2.78; 95% CI, 1.09–7.07; *p* = 0.032).

A large retrospective study from the Netherlands (19) reported 694 cases of CVD among 2,512 TGCT survivors after a median follow-up of 18.4 years (range 5–38.4). The most prevalent diagnoses were coronary artery disease - including 141 cases of myocardial infarction (20.3%) and 150 cases of angina pectoris (21.6%) - followed by peripheral vascular disease (79; 11.4%), heart failure (66; 9.5%), and cerebrovascular accidents (55; 7.9%). Compared to age-matched data from the general Dutch male population, the standardized incidence ratio (SIR) for coronary artery disease was 1.17 (95% CI, 1.04–1.31). The risk of myocardial infarction was significantly higher in nonseminoma survivors under 45 years and those aged 45–54 years (SIR 2.06 and 1.86, respectively), compared to the age-matched general population. An almost two-fold increased risk was also observed across all age groups within 5 to 9 years after treatment (SIR 1.91). However, the risk in this study declined in survivors aged > 54 years and in those followed for 10 years or more. In a subsequent study (20), a higher risk of coronary artery disease was observed in patients who received subdiaphragmatic radiotherapy combined with chemotherapy compared to those treated with chemotherapy alone (SIR 2.3; 95% CI, 1.6–3.1 *vs*. SIR 1.4; 95% CI, 1.0–1.8).

Most data on the incidence of cardiovascular late effects in testicular cancer survivors have been derived from epidemiological studies with follow-up periods of up to 20 years post-treatment. Consequently, limited information is available on the health status of TGCT survivors beyond 20 years after the completion of cancer treatment (15, 26, 31).

A Norwegian study (*N* = 990) with a median follow-up of 19 years (range, 13-28) (21) demonstrated increased risks of atherosclerotic disease in all treatment groups compared to the surgery only: radiotherapy (hazard ratio [HR] 2.3; 95% CI, 1.03–5.3), BEP (bleomycin, etoposide, and cisplatin) chemotherapy (HR 4.7; 95% CI, 1.8–12.2), and radiotherapy + chemotherapy (HR 4.7; 95% CI, 1.6–14.1). Furthermore, BEP chemotherapy was associated with a 3.1-fold increased risk of myocardial infarction (95% CI, 1.2–7.7) compared to age-matched healthy male controls, with an even higher risk observed in the radiotherapy + chemotherapy group (HR 4.8; 95% CI, 1.6–13.9). Notably, the risk of incident stroke was significantly elevated in survivors treated with radiotherapy alone (HR 4.0; 95% CI, 1.4–11.7), while no significant increase was observed in the other treatment groups compared to healthy controls.

A population-based study (22) evaluated short- and long-term CVD mortality in nonseminoma patients treated with chemotherapy (*n* = 6,909) or surgery alone (*n* = 8,097). CVD mortality was significantly increased in the chemotherapy group (standardized mortality ratio [SMR] 1.36; 95% CI, 1.03–1.78), but not in the surgery-only group (SMR 0.81; 95% CI, 0.60–1.07). The excess CVD mortality following chemotherapy was primarily confined to the first year after diagnosis (SMR 5.31; absolute excess risk [AER] 13.90 per 10,000 person-years), and was mainly attributable to cerebrovascular disease (SMR 21.72; AER 7.43) and heart disease (SMR 3.45; AER 6.64).

Consistent with previous findings, Lauritsen et al. (16) reported that among 1,819 patients treated with BEP chemotherapy, there were significantly increased risks for myocardial infarction (HR 6.3; 95% CI, 2.9–13.9), cerebrovascular accident (HR 6.0; 95% CI, 2.6–14.1), and venous thromboembolism (HR 24.7; 95% CI, 14.0–43.6) during the first year after treatment initiation. One year after completing BEP treatment, the risk of CVD returned to levels comparable to the general population. However, after 10 years, an increased risk re-emerged for myocardial infarction (HR 1.4; 95% CI, 1.0–2.0) and cardiovascular-related mortality (HR 1.6; 95% CI, 1.0–2.5).

In a recent study (24) with a median follow-up of 16.1 years (range, 0–38.5), 272 out of 4,748 TGCT patients developed CVD. Among them, 64% experienced a myocardial infarction, and 28% had coronary artery disease without infarction. Subsequently, 16% of these patients developed heart failure. In 6% of cases CVE were fatal (n = 16). Compared to orchiectomy alone, cisplatin-based chemotherapy was associated with an increased risk of CVD (HR 1.9; 95% CI, 1.1–3.1). Additionally, survivors who developed CVD after treatment reported significantly lower quality of life across multiple domains, including physical and social functioning, role limitations due to physical health, less energy and vitality, and had lower general health score compared to survivors without CVD (all *p* ≤ 0.01).

In summary, evidence from multiple studies demonstrates a consistently increased risk of CVD among testicular cancer survivors, particularly following cisplatin-based chemotherapy and radiotherapy. Combined modality treatment appears to be associated with a higher cardiovascular risk than chemotherapy alone. The most commonly reported clinical manifestations include myocardial infarction, angina pectoris, cerebrovascular accident, venous thromboembolism, and heart failure. The risk is highest in the first year after treatment but can persist or recur even after a decade. Furthermore, TGCT survivors who develop CVD report significantly lower quality of life.

## 3 Pathophysiology of cisplatin-related cardiovascular toxicity

Cisplatin-induced CVD involves both vascular and heart damage through several distinct but interconnected mechanisms. Three key hypotheses have been proposed to explain the pathophysiology of CVD in testicular cancer survivors treated with cisplatin-based chemotherapy (33, 34). The direct vascular damage hypothesis postulates that cisplatin directly injures the vascular endothelium, initiating early vascular toxicity. The indirect hypothesis proposes that cisplatin-based chemotherapy increases the prevalence of traditional cardiovascular risk factors - such as hypertension, dyslipidemia, and insulin resistance - which then contribute to long-term cardiovascular morbidity (34). Finally, the multiple-hit hypothesis suggests that combination of direct endothelial injury and chemotherapy-induced risk factor accumulation acts synergistically to increase the overall risk of CVD in this population (33).

### 3.1 Mechanisms of cisplatin-induced vascular toxicity

The mechanisms by which cisplatin may cause arrhythmias and heart failure are not yet completely understood. However, the mechanisms of acute and late vascular toxicity have been more clearly explained. Cisplatin-based regimens used in the treatment of TGCT primarily drive vascular toxicity by endothelial dysfunction and prothrombotic state (30, 31). Platinum-therapy related damage of vasculature may be mediated also through inflammatory response to cytokine release, oxidative stress and electrolyte dysbalance (35).

#### 3.1.1 Endothelial dysfunction

Endothelial dysfunction is characterized by an imbalance between vasodilation and vasoconstriction, shifting toward reduced vasodilation and a proinflammatory, prothrombotic state. In patients receiving cisplatin, impaired endothelium-dependent vasodilation may be attributed to reduced AKT–endothelial nitric oxide synthase (eNOS) signaling. Endothelial dysfunction plays a central role in the cascade of events leading to atherosclerosis and subsequent CVD and cerebrovascular accidents. In addition to classical cardiovascular risk factors - such as dyslipidemia, arterial hypertension, hyperglycemia, smoking, and diabetes mellitus - vascular toxicity is further influenced by chronic inflammation, oxidative stress, and shear stress, all of which reduce the bioavailability of nitric oxide. These mechanisms not only contribute to endothelial dysfunction and vascular injury but may also accelerate vascular aging and increase long-term cardiovascular risk (30–32, 36).

Cisplatin has a direct cytotoxic effect on endothelial cells. Acute and late vascular toxicity induced by cisplatin therapy appear to have distinct pathogenic mechanisms.

In the early phase, vascular toxicity is mainly caused by oxidative stress, endothelial apoptosis, platelet activation, and thrombus formation. Cisplatin-induced oxidative stress is primarily driven by mitochondrial reactive oxygen species (ROS). This oxidative stress leads to mitochondrial damage, including membrane depolarization and ultrastructural abnormalities. It is characterized by excessive ROS generation, lipid peroxidation, depletion of antioxidant defenses, and activation of inflammatory and apoptotic signaling pathways that contribute to adverse effects, such as thromboembolism, myocardial infarction, stroke, and newly diagnosed hypertension (29, 30).

In addition to oxidative stress, inflammation also plays a crucial role in cisplatin-induced endothelial dysfunction. This dysfunction is a complex process involving cytokines such as TNF-α, IL-1β, IL-6, IL-8, IL-18, IL-4, and IL-13; chemokines like RANTES; cell adhesion molecules; transcription factors including NF-κB and STAT6; the NLRP3 inflammasome; endothelins; and other inflammatory mediators.

Long-lasting degenerative changes in vasculature initiated by platinum-based chemotherapy are believed to be key pathogenic drivers of late vascular toxicity (31, 32). Late toxicity is associated with persistent and irreversible endothelial dysfunction, vascular remodeling, and chronic degenerative processes. This contributes to an elevated risk of cardiovascular death, coronary artery disease, myocardial infarction, hypertension, hyperlipidemia, diabetes mellitus, and metabolic syndrome (16, 30–32, 36, 37). Studies in long-term testicular cancer survivors have also demonstrated elevated levels of circulating endothelial cells detectable even two decades after the completion of cancer therapy (38). Notably, circulating cisplatin levels can also remain detectable for many years following treatment (39).

Recent preclinical research has provided new insights into endothelial alterations induced by cisplatin treatment at the molecular level. Using single-cell sequencing, specific gene expression changes were identified, revealing significant upregulation of pathways involved in DNA damage (Ddit4, Acer2), hypoxia (Phlda3, Mt1, Slc3a2, Ier3, Klf9, Adipor2, UCP2), inflammatory responses (Timp4, Tns1, Gdf15, Neat1), cell cycle arrest (Trp53inp1), intrinsic and extrinsic apoptosis (Fas, Bax, Ei24, Tgm2), blood vessel remodeling (Pim3), angiogenesis (Timp3, Flt1), and cellular senescence (Cdkn1a) (40).

#### 3.1.2 Prothrombotic state

Several pathogenetic mechanisms have been proposed to explain how cisplatin-based chemotherapy may contribute to a prothrombotic state, such as:

- direct cytotoxic effect of cisplatin on endothelium;
- the release of procoagulant factors and cytokines from damaged cancer cells;
- decreased production of anticoagulants due to liver damage - either from cancer itself or from chemotherapy-induced hepatotoxicity.

Multiple studies have demonstrated that cisplatin can cause hepatotoxicity in both *in vitro* and *in vivo* models. Regarding the specific impact on production of procoagulant and anticoagulant molecules, there is no direct evidence or clinical studies explicitly demonstrating effects of cisplatin on these pathways in the liver.

Acute thrombosis has been reported during cisplatin therapy even in the absence of atherosclerotic plaque rupture or clinically manifested atherosclerosis (37).

In recent years, superficial erosion has gained attention as a patomechanism underlying acute coronary syndromes in patients treated with cisplatin chemotherapy (32). Moreover, blood flow perturbations may induce endothelial activation and the recruitment of inflammatory cells, particularly neutrophils. Early atherosclerosis is characterized by the formation of fatty streaks, during which oxidized low-density lipoprotein (LDL) particles accumulate in the arterial intima. This triggers a cascade of endothelial inflammation and dysfunction, accompanied by the secretion of chemoattractant molecules that facilitate the recruitment of monocytes and lymphocytes. Concurrently, upregulation of adhesion molecules on endothelial cells promotes the adherence of monocytes and T lymphocytes to the intimal surface, contributing to lesion progression and endothelial apoptosis (41–44). Furthermore, von Willebrand factor (vWF) is released from damaged endothelial cells and secreted into the circulation or subendothelial space. This molecule stimulates platelet adhesion, activation and aggregation (45).

The risk of arterial thromboembolic events appears to be highest during the first month after starting chemotherapy and remains significantly elevated throughout the first year. In addition to acute arterial thromboembolism, both the venous and arterial compartments of the vascular system are involved in thrombotic complications in patients with TGCT (37, 43, 46, 47). Cisplatin may also increase levels of vWF in circulation. Elevated circulating vWF serves as a biomarker of endothelial injury. Nuver et al. (48) were among the first to observe an increase in vWF levels during chemotherapy in patients with TGCT, supporting the hypothesis that chemotherapy induces endothelial damage. Patients with preexisting elevated vWF levels may be at higher risk of cardiovascular toxicity. In addition, the persistent elevation of vWF after treatment suggests ongoing endothelial stimulation or damage following cisplatin-based chemotherapy (37).

### 3.2 Mechanisms of cisplatin-induced cardiac toxicity

Pathogenesis of cisplatin-induced cardiac toxicity is complex and not yet fully understood. However, findings from preclinical studies suggest multiple mechanisms including oxidative stress, mitochondrial damage, inflammatory process, apoptosis, and alterations in cardiac proteins and hemodynamics.

Cisplatin-induced cardiac damage is thought to be primarily exacerbated by oxidative stress. Cisplatin causes excessive production of ROS in cardiac tissue, overwhelming the antioxidant defense systems such as glutathione and superoxide dismutase. This oxidative stress can lead to mitochondrial DNA damage, mitochondrial membrane depolarization, and ultrastructural abnormalities in the mitochondria (28, 44). Cisplatin may accumulate in the mitochondrial matrix, disrupting mitochondrial respiration and depleting intracellular energy levels. Moreover, it may activate signaling pathways such as MAPKs and PI3K/Akt, which are involved in apoptosis in cardiac cells (49).

Cisplatin- related cardiac damage may be mediated through an inflammatory process, Accumulating evidence indicates that cisplatin increases secretion of several pro-inflammatory cytokines and chemokines (such as interleukin-1 and -6, tumor necrosis factor alpha (TNF-α) The translocation of the transcription factor nuclear factor kappa B (NF-κB) from the cytosol to the nucleus promotes the production pro-inflammatory TNF-α in cardiomyocytes (28, 50, 51).

In addition to oxidative stress and inflammation, apoptosis plays a crucial role in cisplatin-induced cardiac toxicity. It has been demonstrated in both *in vivo* and *in vitro* models. Cisplatin induces the release of pro-apoptotic factors such as cytochrome c, endonuclease G, and apoptosis-inducing factor (AIF) from the mitochondria into the cytosol. Cytochrome c activates caspase-9, which in turn triggers a cascade of downstream caspase activation, leading to apoptosis in a caspase-dependent manner. Conversely, endonuclease G and AIF translocate to and accumulate in the nucleus after their mitochondrial release, inducing apoptosis via a caspase-independent pathway (51). Furthermore, cisplatin induces alterations in renal tubular cells functions, which leads to impaired magnesium reabsorption. Hypomagnesemia is associated with an increased incidence or aggravation of hypertension, heart failure, serious cardiac arrhythmias, including torsades de points (52).

**Figure 1:**
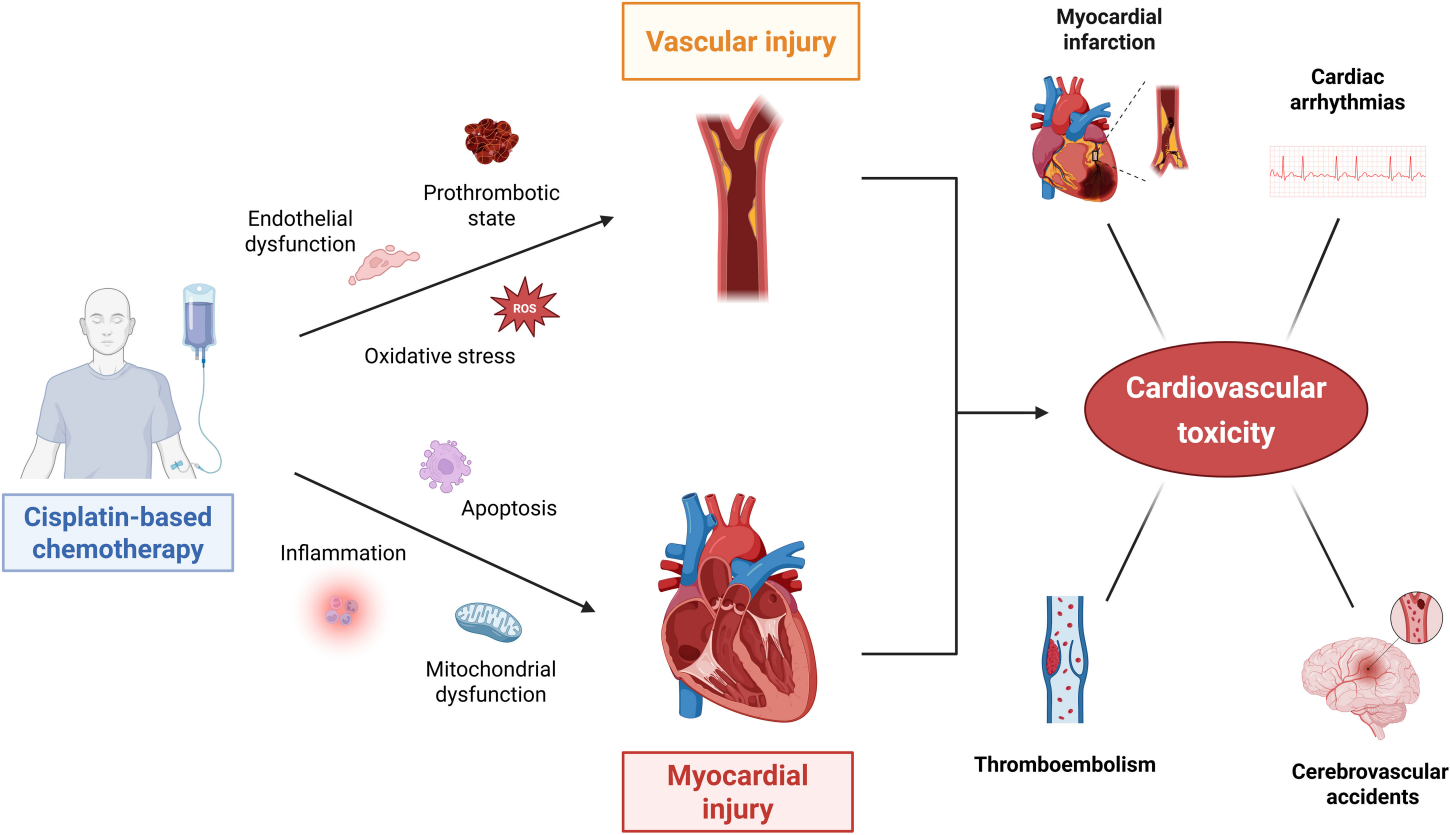
Molecular mechanisms of cisplatin-induced cardiovascular toxicity in testicular germ cell tumor survivors. Cisplatin-based chemotherapy contributes to cardiovascular toxicity through two interconnected pathways: vascular injury and myocardial injury. In the vascular compartment, cisplatin promotes endothelial dysfunction, oxidative stress, and a prothrombotic state, leading to early thrombotic events and long-term development of atherosclerosis. Concurrently, myocardial toxicity is mediated by inflammation, oxidative stress, apoptosis, and mitochondrial dysfunction, resulting in direct cardiomyocyte injury. These processes collectively contribute to increased risks of CVD, such as myocardial infarction, heart failure, arrhythmias, cerebrovascular events, and thromboembolism in testicular cancer survivors. This figure was created using BioRender.com.

Myocardial toxicity is primarily mediated by oxidative stress, inflammation, and apoptosis, resulting in mitochondrial dysfunction and cardiac cell injury. Together, these processes can contribute to the increased cardiovascular risk seen in long-term survivors of testicular cancer treated with cisplatin. Mechanisms of cisplatin-induced cardiovascular toxicity, along with the most common clinical manifestations, are illustrated in **Figure 1**.

## 4 Cardiovascular risk factors

The increased CVD risk observed in TGCT survivors cannot be attributed solely to the direct toxicity of chemotherapy or radiotherapy. Growing evidence indicates a higher prevalence of cardiovascular risk factors - such as smoking, hypertension, dyslipidemia, diabetes mellitus, obesity, and metabolic syndrome - among this population (16–18, 21, 24, 26, 53–61). Selected studies from the past 15 years evaluating cardiovascular risk factors among TGCT survivors treated with cisplatin-based chemotherapy are summarized in **Table 2**.

**Table 2:** Summary of selected studies evaluating cardiovascular risk factors in testicular germ cell tumor survivors treated with cisplatin-based chemotherapy (since 2010).

Characteristics of studies					Prevalence of cardiovascular risk factors compared to controls (%)					
Author (Year)	N	Control group (N)	Medain follow-up, years (range) or mean ± SD	Median age at follow-up, years (range) or mean ± SD	Smoking	Hypertension	Dyslipidemia	Diabetes mellitus	Obesity	Metabolic syndrome
Haugnes et al. (2010) (21)	364	Orchiectomy only (206) and healthy men (990)	19 y (13-28)	51 y (31-69)	56% vs. 56% vs. 59% ^a^	26% vs. 12% vs. 13% ^b^	14% vs. 14% vs. 9% ^c^	5.4% vs. 4% vs. 4.3%	17% vs. 19% vs. 23%	NR
Willemse et al. (2013) (58)	174	Healthy men (360)	5 y (0.1–30.2)	38.7 y (31-48)	both ~40%	31% vs. 22.5%	20.1% vs. 10%	6.3% vs. 4.4%	29.3% vs. 19.4%	16.7% vs. 8.1%
Feldman et al. (2018) (61)	787	Healthy men (787)	4.2 y (1.0-29.9)	37.3 y (18.7-68.4)	8.4% vs. 28.2%	10.9% vs. 10.5%	NR	2.4% vs. 3.8%	NR	NR
Lauritsen et al. (2020) (16)	2,114 ^d^	Healthy men (51,850)	15.8 y (9.8-22.0)	NR	NR	11.9% vs. 13.1% ^b^	7.7% vs. 8.7% ^c^	2.7% vs. 3.1%	NR	NR
Bjerring et al. (2021) (26)	94	Healthy men (94)	29.8 ± 4.1 y	60 ± 9 y	38% vs. 41%	55% vs. 24% ^b^	44% vs. 18% ^c^	11% vs. 4%	23% vs. 18%	NR
Lubberts et al. (2023) (24)	159	Orchiectomy only (69)	16.1 y (0-38.5)	NR	NR	54% vs. 45%	85% vs. 84%	9% vs. 7%	9% vs. 16%	37% vs. 30%

NR, not reported; SD, standard deviation; y, years.

a Includes both current and previous daily smoker.

b Patients taking antihypertensive medication.

c Patients taking lipid-lowering medication.

d 295 survivors received more than one line of therapy.

### 4.1 Smoking

Smoking is a well-known risk factor for CVD and many types of cancer, and remains the leading preventable cause of disease, death, and disability in the United States (62). According to data from the 1992–2023 National Health Interview Survey, 11.4% of cancer survivors aged 18 and older reported current cigarette smoking (63). In TGCT patients, two studies have demonstrated that smoking is associated with worse outcomes and survival in both stage I and metastatic disease (64, 65). The reported prevalence of smoking among TGCT survivors varies in the literature, ranging from 8.4% to 40% (18, 21, 26, 56, 58, 61). In most studies, no significant differences in smoking status were observed between TGCT survivors and the general population, or between different treatment groups (17, 18, 26, 58).

### 4.2 Hypertension

Although the definition of arterial hypertension varied across studies, its high prevalence among TGCT survivors appears indisputable. Over 25 years ago, Meinardi et al. (17) reported a hypertension prevalence of 39% in survivors treated with cisplatin-based chemotherapy, significantly higher than in those with stage I disease treated with orchiectomy alone (13%). A large study (56) evaluating blood pressure in 1,289 TGCT survivors treated with different modalities found a higher prevalence of hypertension in patients who received chemotherapy or radiotherapy compared to those who underwent surgery alone, after a median follow-up of 11.2 years (range, 5–22). Moreover, hypertension rates increased with cumulative cisplatin dose: 39% in the surgery group, 50% in those receiving ≤ 850 mg of cisplatin, and 53% in those receiving > 850 mg (*p* < 0.001). Compared to healthy controls, TGCT survivors who received more than 850 mg of cisplatin had over twice the risk of hypertension (age-adjusted OR 2.3; 95% CI, 1.5–3.7). Notably, the prevalence of hypertension was also high among patients treated with radiotherapy, reaching 54%. The increased odds of hypertension with higher cumulative doses of cisplatin have been confirmed by other studies (16, 57). Furthermore, Beitzen-Heineke et al. (15) demonstrated that a cumulative cisplatin dose ≥ 200 mg/m² was associated with attenuated biventricular systolic function and myocardial tissue alterations in asymptomatic long-term TGCT survivors. Lauritsen et al. (16) reported a persistently elevated risk of hypertension in patients treated with BEP and those receiving more than one line of therapy (HR 1.4, 95% CI, 1.3–1.6; and HR 1.7, 95% CI, 1.3–2.3, respectively), from treatment initiation through the end of follow-up, compared with the general population. In contrast, radiotherapy was not associated with a significantly increased risk of hypertension. Another study (21) found no significant differences in systolic or diastolic blood pressure between treatment groups. However, the use of antihypertensive medication was highest among patients treated with chemotherapy alone (OR 3.1; 95% CI, 1.9–5.2) and with combined chemotherapy and radiotherapy (OR 3.7; 95% CI, 1.6–8.9). In contrast, the radiotherapy-only group did not differ significantly from the surgery group (OR 1.5; 95% CI, 0.9–2.5). Additionally, all cytotoxic treatment groups showed significantly higher prevalences of antihypertensive medication use compared to the general population.

### 4.3 Dyslipidemia

Dyslipidemia is defined as abnormalities in serum lipid levels, including elevated total cholesterol (TC), high triglycerides (TG), low high-density lipoprotein cholesterol (HDL), and elevated low-density lipoprotein cholesterol (LDL) (66). A growing number of studies have reported a high prevalence of lipid abnormalities and the use of lipid-lowering medications among TGCT survivors (17, 21, 24, 26, 53, 55, 58). Haugnes et al. reported that all treatment groups had increased odds of using lipid-lowering medications compared to the healthy male population, with the highest odds observed in the chemotherapy plus radiotherapy group (odds ratio [OR] = 2.59) and the chemotherapy group (OR = 2.07) (21). Another more recent study also showed that the prevalence of lipid-lowering medication use was significantly higher in TGCT survivors compared to healthy controls (44% *vs*. 18%) (26). Interestingly, in a large study by Lubberts et al., the prevalence of dyslipidemia ranged from 84% to 88% and did not significantly differ among the chemotherapy, radiotherapy, and orchiectomy-only groups (*p* = 0.507) (24). However, Willemse et al. reported a higher prevalence of dyslipidemia among patients treated with combination chemotherapy compared to those who underwent surgery alone and to healthy controls (20.1% *vs*. 12.3% *vs*. 10%) (58). In our recent study (67) of a Slovak population of long-term TGCT survivors (N = 154) with a median follow-up of 10 years (range, 4–32), 57.8% had elevated total cholesterol, 42.9% had elevated triglycerides, 10.4% had suboptimal HDL, 72.1% had elevated LDL, and 42.9% had elevated VLDL levels. While lipid profiles did not significantly differ across treatment groups, patients treated with chemotherapy had the highest levels of total cholesterol, triglycerides, LDL, and VLDL, as well as the lowest HDL levels. Moreover, survivors who received cumulative cisplatin doses ≥ 400 mg/m² demonstrated higher total cholesterol, triglycerides, LDL, and VLDL levels, and lower HDL compared to the surveillance group. Despite the lipid abnormalities, the use of lipid-lowering medications in our population was surprisingly low (only 3.9%).

### 4.4 Diabetes mellitus

Diabetes mellitus has been observed particularly in long-term TGCT survivors treated with radiotherapy to the retroperitoneal lymph nodes (16, 21). In a study by Haugnes et al. (21), the overall prevalence of diabetes was 7.3% at a median follow-up of 19 years (range, 13–28), with the highest rates seen in the radiotherapy group (10.2%) and the combined chemotherapy plus radiotherapy group (15.6%). Compared to the surgery-only group, the odds ratios were 2.3 (95% CI, 1.5–3.7) for radiotherapy alone and 3.9 (95% CI, 1.4–10.9) for combined treatment. The authors noted that standard dog-leg and para-aortic radiation fields often include most of the pancreatic gland, suggesting that radiation-induced pancreatic dysfunction, including diabetes, may be a long-term complication of infradiaphragmatic radiotherapy (68, 69). Lauritsen et al. (16) reported an increased risk of diabetes more than 10 years after radiotherapy, with HR of 1.4 (95% CI, 1.0–2.0).

### 4.5 Overweight and obesity

People living with overweight or obesity are at increased risk for cardiovascular morbidity and mortality (70). In a recent study (24) of 304 TGCT survivors with a median follow-up of 21.8 years (range, 7.9–39.0), the prevalence of overweight and obesity was 64.5% and 11.5%, respectively. Furthermore, obesity at the time of testicular cancer diagnosis was identified as a significant risk factor for developing CVD after treatment (HR 4.7; 95% CI, 2.4–9.3). The authors noted that, in addition to being an established independent cardiovascular risk factor, adipose tissue may serve as a long-term reservoir for platinum. This suggests that obesity at diagnosis could be associated with prolonged circulating platinum levels following chemotherapy (24). Willemse et al. (58) reported that TGCT survivors had a significantly higher prevalence of obesity and dyslipidemia compared with age-matched healthy controls. Notably, those treated with combination chemotherapy had the highest prevalence (OR 1.7; 95% CI, 1.1–2.6). Interestingly, in a cohort of 455 TGCT survivors treated with cisplatin (median follow-up 26 months; IQR 16–59 months), a higher visceral-to-subcutaneous fat ratio - measured using pre-chemotherapy CT scans - was significantly associated with an increased risk of developing cardiometabolic conditions such as hypertension, dyslipidemia, or diabetes among obese men (age-adjusted HR 3.14; 95% CI, 1.02–9.71). The study also found that post-chemotherapy weight gain was primarily due to visceral fat accumulation, which correlated with higher estimated cardiovascular risk, especially in younger men. These findings support central obesity as a key predictor of cardiometabolic complications in this population (71).

### 4.6 Metabolic syndrome

Metabolic syndrome is defined as a combination of abdominal obesity, hypertension, dyslipidemia, and insulin resistance that together significantly increase the risk of CVD (72–75). Over the past decades, several studies have evaluated the risk of metabolic syndrome among TGCT survivors (21, 55, 57, 58, 60, 76–78). Furthermore, several studies have reported an association between low testosterone levels and the development of metabolic syndrome in this population (55, 60, 76). However, reported prevalence rates vary due to differences in the definition of metabolic syndrome, characteristics of control groups, and length of follow-up. While many studies have demonstrated an increased prevalence of individual components of metabolic syndrome in TGCT survivors, the overall evidence remains inconclusive regarding an increased prevalence of metabolic syndrome itself after cancer treatment. Some studies have found an association with cisplatin-based chemotherapy (55, 58, 76), whereas others have not (57, 60, 78).

### 4.7 Hypogonadism

In aging men, hypogonadism, or testosterone deficiency, is associated with a range of complications, including an increased risk of osteoporosis, metabolic syndrome, obesity, diabetes, and CVD. In addition, it is also associated with decreased quality of life (79, 80). In testicular cancer patients, hypogonadism may develop following any treatment modality (10). There is strong evidence that chemotherapy, higher cumulative doses of cisplatin, infradiaphragmatic radiotherapy, and combination of chemotherapy and radiotherapy are associated with an increased risk of testosterone deficiency in TGCT patients compared to those treated with orchiectomy alone. The risk appears to be highest among patients who received higher doses of cisplatin and combination of chemotherapy and radiotherapy (81). A recent study by Fosså et al. reported that 40% of TGCT survivors over the age of 60 had low testosterone levels, compared to 10% of age-matched healthy controls, after a median follow-up of 27 years (range 24–31 years). Nearly three decades after testicular cancer diagnosis, the probability of biochemical hypogonadism was significantly associated with advancing age and higher treatment intensity (82). As mentioned earlier, several studies have identified low testosterone levels a potential risk factor of metabolic syndrome in TGCT survivors (55, 60, 76). Bogefors et al. reported that testicular cancer survivors with hypogonadism had significantly higher insulin levels compared to eugonadal patients. Additionally, the hypogonadal group had an increased risk of metabolic syndrome (OR = 4.4; *p* = 0.01) (60). Results from the Platinum Study (83) showed that among 491 testicular cancer survivors with median age 38.2 years (range 18.7 - 68.4 years), 38.5% had hypogonadism. Compared to survivors without hypogonadism, those with hypogonadism were more likely to use lipid-lowering medications (20.1% *vs*. 6.0%; *p* < 0.001) or antihypertensive medications (18.5% *vs*. 10.6%; *p* = 0.013). A marginally significant trend was also observed for increased use of medications for diabetes (5.8% *vs*. 2.6%; *p* = 0.07).

To summarize, the available evidence indicates that testicular cancer survivors, especially those treated with cisplatin-based chemotherapy or radiotherapy, show a consistently higher prevalence of cardiovascular risk factors. These include hypertension, dyslipidemia, diabetes, and obesity. Hypertension and lipid abnormalities are among the most frequent findings, often linked to higher cumulative cisplatin doses. Diabetes is more common in survivors treated with retroperitoneal radiotherapy, likely due to pancreatic exposure. Obesity - particularly central obesity - and weight gain after treatment are also key contributors and may be related to persistent platinum exposure. Importantly, hypogonadism has emerged as a significant and prevalent long-term complication, affecting over a third of survivors, and is strongly associated with many of the adverse health outcomes. Testosterone deficiency contributes to the development of metabolic syndrome, insulin resistance, obesity, and dyslipidemia, and it may further exacerbate CVD risk in this population.

## 5 Discussion

This review synthesizes current evidence on cardiovascular toxicity in testicular cancer survivors, emphasizing the complexity of cardiovascular disease in this unique population. While the cardiotoxic effects of cisplatin-based chemotherapy and retroperitoneal radiotherapy are established contributors to increased CVD risk, it is clear that treatment-related toxicity alone does not fully explain the elevated incidence of cardiovascular morbidity. The high prevalence of traditional cardiovascular risk factors - such as hypertension, dyslipidemia, diabetes mellitus, obesity, and metabolic syndrome - among TGCT survivors underscores a multifactorial etiology involving both direct treatment effects and lifestyle or metabolic influences.

With the growing number of testicular cancer survivors worldwide, there is an increasing need for a structured approach to survivorship care. Given the high prevalence of cardiovascular risk factors among this population, effective long-term strategies for monitoring and prevention of CVD are essential. Early identification of treatment-related toxicities and integration of preventive care along with surveillance for recurrence should be central components of survivorship management (84).

Cardiovascular complications may develop years or even decades after cancer treatment, often progressing silently before becoming clinically apparent. Therefore, long-term follow-up is critical to identify early signs of cardiometabolic dysfunction. Based on current evidence, regular assessment of blood pressure, lipid profiles, hormone and plasma glucose levels, and body composition should be integrated into survivorship care plans - especially for testicular cancer survivors who have received cisplatin-based chemotherapy or radiotherapy to the retroperitoneum (85). All cancer survivors, including those with a history of testicular cancer, are advised to undergo annual clinical evaluations and optimization of cardiovascular risk factors. Individuals at higher risk should receive additional cardiovascular assessments, such as cardiac imaging. Routine testing for biomarkers to detect early cardiovascular damage is not currently recommended (86).

Effective prevention and management of CVD in testicular cancer survivors require a coordinated, multidisciplinary approach. Oncologists play a central role in identifying patients at risk based on treatment history - particularly those who had advanced disease and those treated with more than one line of conventional-dose chemotherapy and/or high-dose chemotherapy followed by autologous stem cell transplantation. Primary care providers and cardiologists are essential for long-term follow-up, cardiovascular screening, and risk management. Involving other specialists, such as dietitians and physiotherapists, can further support patients in managing metabolic complications and maintaining a healthy lifestyle. Establishing survivorship clinics or care pathways that facilitate communication among specialists can enhance care coordination and improve long-term outcomes (87).

According to the 2022 ESC Guidelines on cardio-oncology, testicular cancer survivors treated with platinum-based chemotherapy should have their cardiovascular risk factors closely monitored and be educated to promptly report any new cardiac symptoms to their healthcare provider. However, the role of screening for coronary artery disease in these patients remains unknown, and predicting individual cardiovascular risk continues to be challenging (88).

One of the most effective strategies for preventing CVD in cancer survivors is regular physical exercise. Over the past decade, numerous studies in testicular cancer survivors have reported the benefits of physical activity in mitigating cancer treatment-related toxicities, improving overall health and quality of life, and even reducing mortality risk (89–94). In addition, structured exercise programs tailored to the needs of cancer survivors can improve functional capacity, muscle strength, and psychological well-being. Given these benefits, incorporating physical activity into survivorship care plans is strongly recommended as part of a comprehensive approach to reduce long-term cardiovascular complications and promote healthy aging in this population (84, 95, 96). Alongside regular physical activity and dietary strategies, 2025 NCCN Survivorship Guidelines recommend counseling cancer survivors on smoking cessation, alcohol moderation, sleep hygiene, and stress management (97).

Despite advances in the field of survivorship care, significant gaps remain in our understanding of cardiovascular toxicity among TGCT survivors:

1. The molecular mechanisms underlying cisplatin-induced vascular and cardiac toxicity are complex and not yet fully understood.
2. The majority of available clinical studies are observational and vary widely in design, treatment exposures, follow-up duration, and outcome definitions, limiting both comparability and the ability to perform robust meta-analyses.
3. The predictive value of existing cardiovascular risk assessment tools, primarily developed for the general population, is unclear in TGCT survivors, who may have unique risk profiles influenced by treatment-related factors.
4. The optimal type, frequency, and intensity of cardiovascular screening, as well as its cost-effectiveness in survivors treated with platinum-based chemotherapy, remain unknown.
5. There is a lack of validated biomarkers and imaging modalities for the early detection of subclinical cardiac injury, hindering opportunities for early intervention.
6. Psychological and socioeconomic determinants that affect adherence to cardiovascular preventive strategies in this population are underexplored.

To address these research gaps, we propose several future research directions to advance the understanding and management of cardiovascular toxicity in TGCT survivors. Preclinical and translational studies are needed to clarify the molecular and cellular mechanisms of cisplatin-induced vascular and myocardial damage. To improve comparability and facilitate synthesis of findings, future clinical research should prioritize prospective, multicenter cohort studies with standardized protocols for treatment classification, cardiovascular outcome definitions, and follow-up intervals. Collaborative efforts to harmonize data collection would further support pooled analyses and meta-analyses.

**Figure 2:**
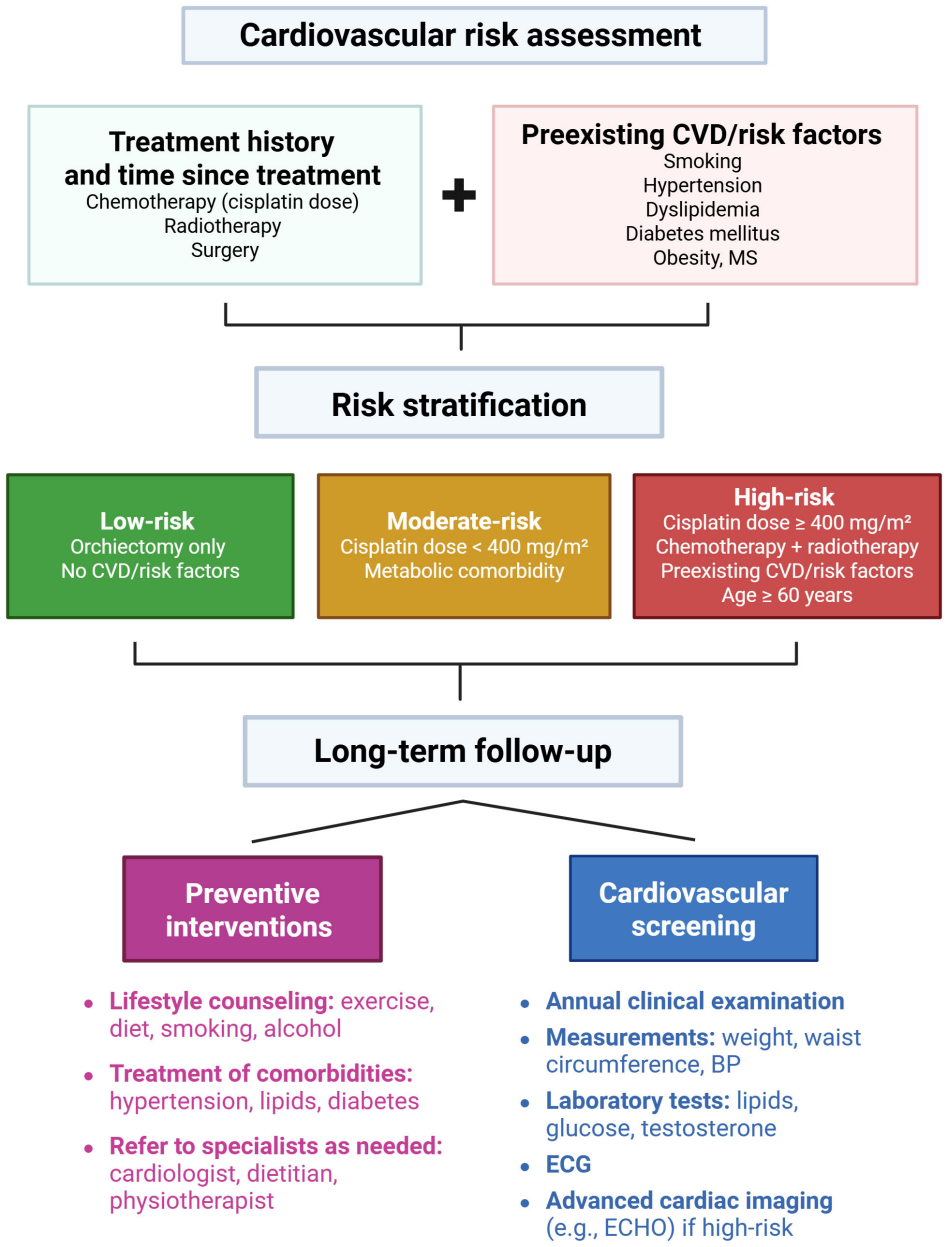
Proposed algorithm for cardiovascular risk assessment and follow-up in testicular cancer survivors. Cardiovascular risk assessment is based on patients’ treatment history, time since treatment completion, and presence of preexisting cardiovascular disease or risk factors. Patients are stratified into low, moderate, and high-risk groups. All patients receive long-term follow-up that includes: (1) preventive interventions such as lifestyle counseling, management of comorbidities, and referral to specialists as needed; and (2) cardiovascular screening, comprising annual clinical examinations, measurements of weight, waist circumference, blood pressure, laboratory tests, ECG, and advanced cardiac imaging when indicated. BP, blood pressure; CVD, cardiovascular disease; ECG, electrocardiogram; ECHO, echocardiography; MS, metabolic syndrome. This figure was created using BioRender.com.

There is a pressing need to develop and validate cardiovascular risk assessment models specifically tailored to TGCT survivors, in order to enhance risk stratification and guide personalized follow-up strategies. A proposed algorithm for cardiovascular risk assessment and follow-up in testicular cancer survivors is outlined in **Figure 2**. Randomized trials or modeling studies should evaluate the effectiveness, optimal timing, and cost-effectiveness of various cardiovascular screening modalities (e.g., echocardiography, arterial stiffness measurements, coronary calcium scoring) in this population. Research should also investigate whether intensified surveillance improves long-term cardiovascular outcomes in high-risk subgroups. There is also a need for clinical trials evaluating interventions such as statins or ACE inhibitors in high-risk survivors. High-risk subgroups can be defined as patients treated with cisplatin-based chemotherapy, receiving a cumulative cisplatin dose of ≥ 400 mg/m² (i.e., undergoing more than one line of chemotherapy), patients treated with both chemotherapy and radiotherapy, and individuals with preexisting cardiovascular risk factors (e.g., hypertension, dyslipidemia, smoking, obesity, diabetes) or diagnosed at an older age. These patients might be more likely to benefit from targeted cardiovascular screening and early lifestyle or pharmacologic interventions.

Furthermore, studies aimed at identifying reliable circulating biomarkers (e.g., cardiac troponins, natriuretic peptides, inflammatory markers, endothelial dysfunction markers) and advanced imaging techniques (e.g., cardiac MRI, strain echocardiography) for the early detection of subclinical cardiac injury are essential. Finally, future research should explore the psychological, social, and economic barriers that influence adherence to cardiovascular prevention guidelines among TGCT survivors, to inform the development of targeted behavioral interventions.

## 6 Conclusion

Testicular cancer survivors face a substantially increased risk of cardiovascular disease due to the combined effects of cancer therapy, particularly cisplatin-based chemotherapy, and modifiable cardiovascular risk factors. Protecting their long-term cardiovascular health requires a proactive, individualized, and multidisciplinary approach. While survival rates are excellent, the risk of late-onset cardiovascular complications remains a significant concern. Integrating cardiometabolic monitoring, lifestyle interventions, and personalized risk assessment into survivorship care is essential. Addressing current evidence gaps through high-quality research will be key to refining screening strategies and improving outcomes. As a young and growing survivor population, testicular cancer survivors deserve focused, sustained efforts to ensure that the success of cancer treatment is not compromised by preventable cardiovascular disease.
